# Endothelial Cell-derived Extracellular Vesicles Size-dependently Exert Procoagulant Activity Detected by Thromboelastometry

**DOI:** 10.1038/s41598-017-03159-0

**Published:** 2017-06-16

**Authors:** Wolfgang Holnthoner, Cornelia Bonstingl, Carina Hromada, Severin Muehleder, Johannes Zipperle, Stefan Stojkovic, Heinz Redl, Johann Wojta, Herbert Schöchl, Johannes Grillari, Sylvia Weilner, Christoph J. Schlimp

**Affiliations:** 1 Ludwig Boltzmann Institute for Experimental and Clinical Traumatology, AUVA Research Centre, Vienna, Austria; 2 Austrian Cluster for Tissue Regeneration, Vienna, Austria; 3 Department of Internal Medicine II, Medical University of Vienna, Vienna, Austria; 4 Ludwig Boltzmann Cluster for Cardiovascular Research, Vienna, Austria; 5 Evercyte GmbH, Vienna, Austria; 6 Christian Doppler Laboratory on Biotechnology of Skin Ageing, Department of Biotechnology, University of Natural Resources and Life Sciences, Vienna, Austria; 7 AUVA Trauma Hospital Salzburg, Academic Teaching Hospital of the Paracelsus Medical University, Salzburg, Austria; 8 Department of Anaesthesia, Critical Care and Pain Medicine, Royal Infirmary of Edinburgh, Scotland, United Kingdom

## Abstract

Endothelial cells (ECs) are major modulators of hemostasis by expressing and releasing pro- and anticoagulant mediators into the circulation. Previous studies showed that cultured ECs release procoagulant mediators into cell culture supernatants as evidenced by the reduction of viscoelastic clotting time. This effect was reversed with an anti-tissue factor antibody. Here, we aimed to investigate whether tissue factor (TF) was released by endothelial-derived extracellular vesicles (EVs) and which portion of the released vesicles displays the most prominent procoagulant properties. After stimulation of ECs with tumor-necrosis factor-α (TNF-α) the supernatants of EC cultures were subjected to differential centrifugation steps to collect larger and smaller EVs which were then characterised by nanoparticle tracking analysis (NTA) and flow cytometry. Mixed with fresh human blood and analysed by thromboelastometry EVs exerted a significant procoagulant stimulus, which could be partly reversed by addition of an anti-TF antibody. Moreover, TF activity was confirmed in the centrifuged fractions. In summary, our results provide evidence of the procoagulant potential of smaller and larger endothelial-derived EV fractions detected by thromboelastometry. The observed effect is most likely due to the release of TF-bearing EVs of different dimensions, which are released upon TNF-α stimulation of endothelial cell cultures.

## Introduction

The endothelium modulates blood coagulation by producing coagulant and anticoagulant factors to maintain the sensitive balance between blood fluidity and its stagnancy^1^. In the classical coagulation cascade model of secondary haemostasis, the initiation of coagulation is triggered via contact activation with negatively charged surfaces (intrinsic pathway) or tissue factor (extrinsic pathway). However it has been proposed by Hoffmann and Monroe that coagulation occurs not as a “cascade”, but in three overlapping stages starting with “initiation”, which occurs on tissue factor (TF) bearing cells, then “amplification”, in which platelets and cofactors are activated to set the stage for large scale thrombin generation, and “propagation”, in which large amounts of thrombin are generated on the platelet surface. The authors also state that although endothelial cells express active antithrombotic features preventing coagulation from being initiated in the intact endothelium, inflammatory cytokines can also induce endothelial cells to increase expression of TF and surface adhesion molecules as an adaptive defence mechanism facilitating hemostasis at sites of injury^2^. In addition, it has been recently demonstrated that extracellular vesicles (EVs) play a relevant role in coagulation^3^. Moreover, in work already published ~50 years ago Wolf found that platelet derived EVs (termed “platelet dust”) contribute to blood coagulation^4^.

EVs are small vesicles, consisting of a lipid bilayer membrane which contain proteins, and coding as well as non-coding RNA and DNA^5^. Primarily based on their size and the site of cellular origin, three different types of EVs are distinguished: (I) Shedding microvesicles which directly bleb from the plasma membrane (<1000 nm in diameter), (II) exosomes derived from the endosomal compartment (approx. 30–150 nm in diameter) and (III) apoptotic bodies which are released in the late stage of apoptosis (>1000 nm in diameter)^6^. However, current isolation methods including differential centrifugation are not able to fully separate different types of EVs from each other^7^. Therefore, the collective term “extracellular vesicles” is used. Interestingly, EVs were shown to play an important role in cell to cell communication in physiological^8, 9^ as well as pathological conditions such as cardiovascular disease^7^ and the formation of atherosclerotic plaques^10^.

TF is a 47 kD sized transmembrane protein. It’s primary function is to activate the coagulation process via Ca^2+^-dependent factor VII activation. Besides transmembrane bound TF, a non-cell-associated isoform has been described, which is involved in thrombus formation and thus might contribute to pro-thrombotic states that are found in a number of pathological procoagulant conditions^11^.

Previous studies have shown that endothelial cells (ECs) release EVs exposing TF on their surface^12^. Moreover, it has been reported that tumor necrosis factor-α (TNF-α) increases blood levels of EC-derived microparticles in mice^13^. In addition, shear stress has been reported to regulate the release of endothelial microparticles^14, 15^. Besides their clinical application to detect coagulopathy during the management of bleeding patients, viscoelastic tests such as thromboelastometry (ROTEM) have recently come into focus to investigate procoagulant patterns^16, 17^. In our previous study we demonstrated that ECs release procoagulant mediators into cell culture supernatants as evidenced by the reduction of whole blood clotting time in thromboelastometry. This effect can be partially reversed with an anti-TF antibody^18^. However, the influence of different fractions of endothelial-derived EVs on viscoelastic clotting parameters has not been investigated thus far. In the current study, we aimed to test different subsets of endothelium-derived EVs with regard to their coagulatory activity and whether this effect is mediated by TF. In order to evaluate this hypothesis, we characterised different fractions obtained by differential centrifugation with nanoparticle tracking analysis (NTA) and flow cytometry. Finally, we functionally analyzed the fractions by ROTEM and a TF activity assay.

## Results

### Characterisation of P14 and P100 fractions

**Figure 1:**
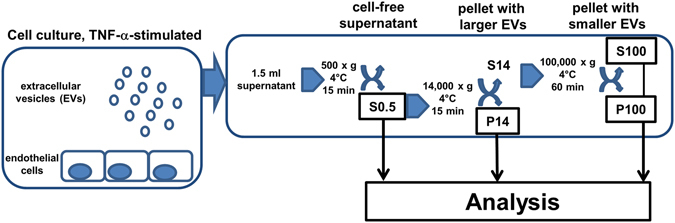
Differential centrifugation of EC culture supernatants. In order to remove the residual cells and cell debris, cell culture supernatants from TNF-α-stimulated HUVEC were initially centrifuged at 500× g to gain a cell free supernatant (S0.5). Thereafter, centrifugation of the S0.5 at 14,000× g pelleted the larger vesicles such as shedding microvesicles and apoptotic bodies (P14), while smaller EVs remained in the supernatant. Finally, this supernatant was subjected to ultracentifugation at 100,000× g, resulting in a pellet (P100) containing EVs enriched in exosomes and a supernatant free of vesicles (S100).

**Figure 2:**
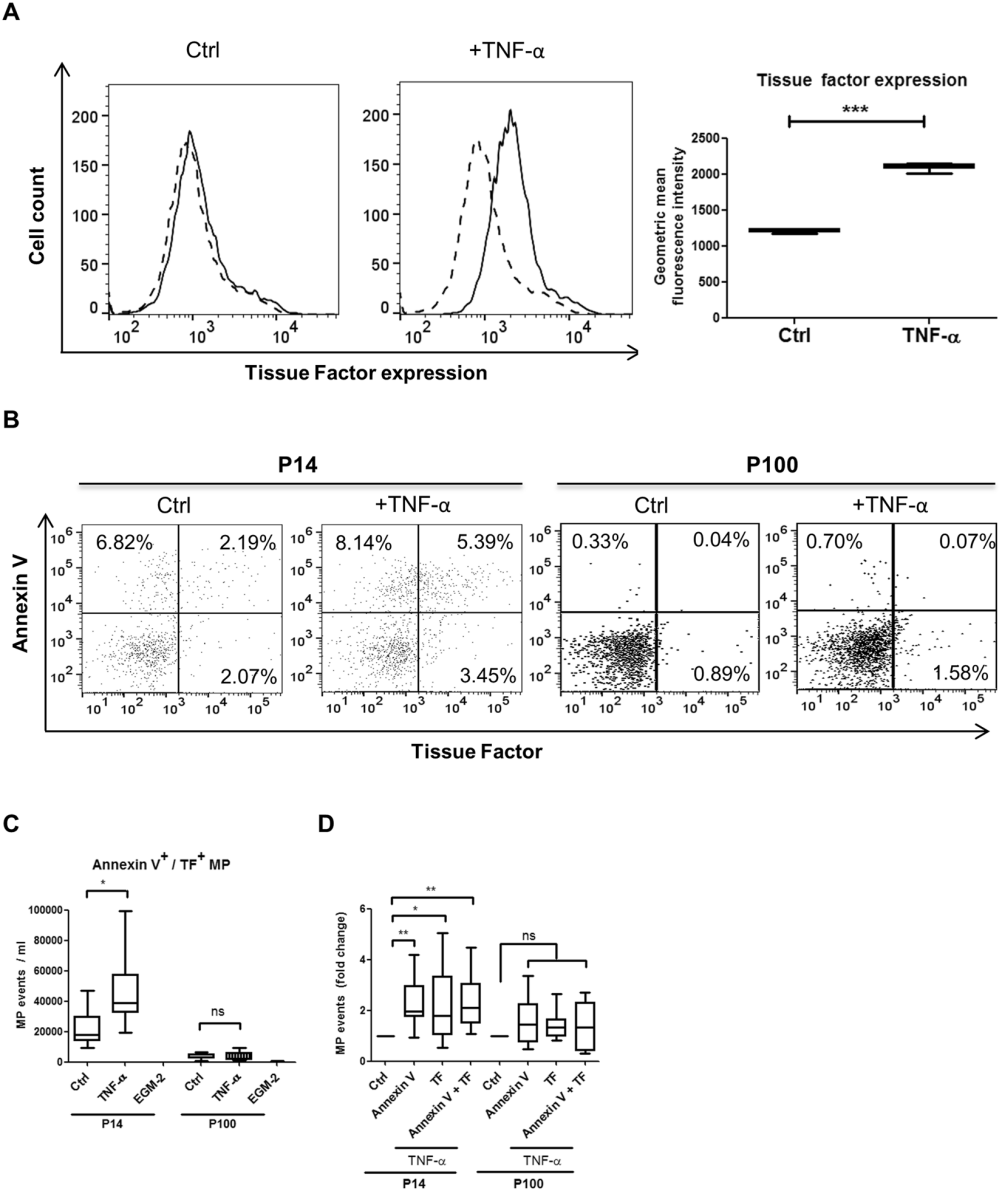
Characterisation of endothelial EVs by flow cyometry and nanoparticle tracking analysis. TF expression on the cell surface is increased upon stimulation of HUVEC with 10 ng/ml TNF-α as measured by flow cytometry; n = 6 (**A**). Representative flow cytometric dot plots of Annexin V^+^/TF^+^ EVs obtained from processed HUVEC culture supernatants are shown. TNF-α stimulation increased the number of Annexin V^+^/TF^+^ events in P14 while this effect could not be observed in P100 (**B**). Quantification of flow cytometry data reveals a significant increase in Annexin V^+^/TF^+^ particle numbers in P14 after stimulation with TNF-α while no significant differences in P100 could be found. EGM-2 was incubated without cells to serve as a negative control and did not contain any Annexin V^+^/TF^+^ particles in neither fraction (**C**). Fold change analyses of single- and double-positive fractions show that Annexin V^+^, TF^+^ and Annexin V^+^/TF^+^ particles in P14 are significantly increased after TNF-α stimulation while there is no significant difference in any fraction of P100 (**D**). n = 6 (**A**); n = 9 (**C**,**D**); Ctrl = control (non TNF-α–stimulated samples); P14 = pellet containing larger EVs; P100 = pellet containing smaller EVs; EGM-2 = endothelial growth medium-2. ns = not significant; *p < 0.05; ** < 0.01; ***p < 0.001.

We have previously shown a TF-dependent reduction of clotting time (CT) in the otherwise non-activated ROTEM assay when adding TNF-α-stimulated endothelial cell culture supernatants to whole blood from healthy volunteers^18^. Since we attributed this effect to endothelial EVs, we aimed to isolate and characterise the different fractions of EVs which caused the observed biological effects. Subsequently, we measured TF expression on cells and on particles released thereof, as outlined in Fig. 1. Upon TNF-α-stimulation for 24 h, we detected a significantly increased TF expression on human umbilical vein endothelial cells (HUVEC) (Fig. 2A). Next we aimed to isolate and characterise EVs on TNF-α-stimulated and non-stimulated HUVEC. To detect particles smaller than 1 µm in cell culture supernatants using flow cytometry, we used silica beads of defined sizes (Supp. Fig. 1). We isolated the P14 (pellet obtained after 14,000× g centrifugation) and P100 (pellet obtained after 100,000× g centrifugation) fractions from cell culture supernatant and analysed the particle count. Supernatant from TNF-α stimulated HUVEC showed an increase in Annexin V^+^/TF^+^ particles in P14 fractions while only few particles could be detected in P100 (Fig. 2B). Quantification of flow cytometry data showed a significant increase in particle concentration upon TNF-α stimulation in P14 while no significant difference could be seen in P100 (Fig. 2C). Furthermore, we found that both Annexin V^+^/TF^+^ and single positive particles were significantly elevated in P14 TNF-α-stimulated samples compared to the respective unstimulated controls while no significant change could be detected in P100 samples (Fig. 2D). To analyse small particles obtained in the fractions that are undetectable with flow cytometry, we employed nanoparticle tracking analysis (NTA). We found that the average size of particles obtained in the P14 fraction was 89 nm in controls and 90 nm in TNF-α-stimulated samples and the average particle count was 7.85 × 10^8^ and 5.87 × 10^8^ particles/ml for control and TNF-α-stimulated samples, respectively. Furthermore, the average size of particles obtained in the P100 fraction was 83 nm in both controls and TNF-α-stimulated samples and the average concentration is 7.2 × 10^8^ and 6.9 × 10^8^ particles/ml for control and TNF-α-stimulated samples, respectively (Suppl. Fig. 4A,B). No significant difference between control and TNF-α stimulated samples could be determined in P14 or P100 fractions. We also observed that S0.5 and S14 contain similar concentrations of particles while particle counts are significantly decreased in S100 compared to S0.5 and S14 fractions (Suppl. Fig. 4A). No differences in particle size could be observed between S0.5, S14 and S100 or between P14 and P100 (Suppl. Fig. 4B).

### Fractionated EVs change parameters in ROTEM

**Figure 3:**
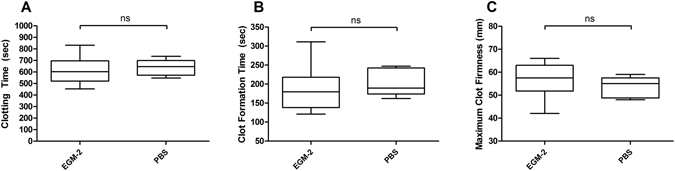
Effect of cell culture medium EGM-2 and sample buffer PBS on ROTEM parameters. For control and exclusion of effects of medium/buffer 50 µl of EGM-2 or PBS were added to 300 µl fresh whole blood and subjected to ROTEM analysis. Box-and-whisker plots (min, max, 25th–75th percentile, median); ns = not significant. n = 4–6 measurements.

**Figure 4:**
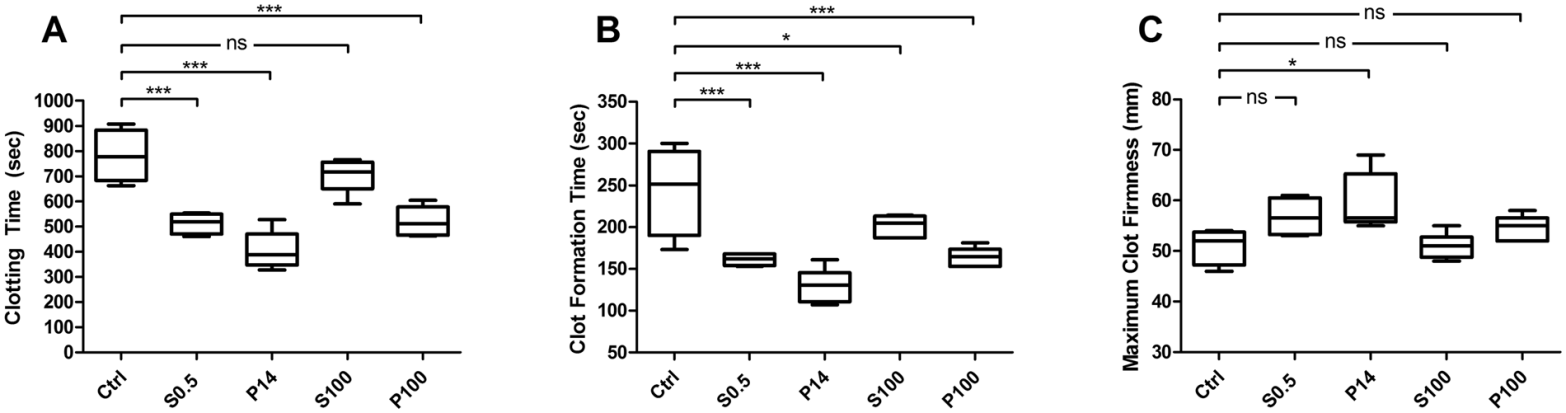
Influence of stimulated EC culture supernatants and concentrated pellets on ROTEM. Different fractions of the supernatants from ECs stimulated with TNF-α for 24 h were collected and 50 µl study samples were added to 300 µl fresh whole blood and subjected to ROTEM analysis. Ctrl = control (phosphate buffered saline); S0.5 = cell free culture supernatant; P14 = pellet containing larger EVs; P100 = pellet containing smaller EVs; S100 = vesicle free supernatant, Box-and-whisker plots (min, max, 25th–75th percentile, median); ns = not significant; *p < 0.05; ***p < 0.001. n = 4–6 measurements.

To confirm the biological effect of isolated particles from TNF-α-stimulated EC culture supernatants we used the ROTEM (NATEM) assay. In order to prove that the cell culture medium itself does not influence the study results, we first compared unconditioned EGM-2 and PBS and did not find any significant differences in the ROTEM parameters CT (clotting time), CFT (clot formation time) and MCF (maximum clot firmness; Fig. 3). We then subjected the different fractions isolated from conditioned media of TNF-α-stimulated ECs to ROTEM analysis (Fig. 4). The cell-free supernatant S0.5 significantly reduced CT in comparison to the control PBS (by a mean of 34%). The vesicle pellets P14 and P100 demonstrated a significant CT-reduction as well (by a mean of 48% and 33%, respectively). In contrast, the largely vesicle-free S100 did not significantly influence the CT (Fig. 4A). Moreover, similar results were obtained for the CFT (reductions by means of 34%, 47% and 33% in S0.5, P14 and P100, respectively; Fig. 4B). Finally, the MCF was slightly increased in the P14 fraction (by a mean of 17%), confirming also the stronger influence of P14 on clot formation/strength, whereas the exposure to fractions S0.5, S100 and P100 did not lead to significant changes in clot strength (Fig. 4C).

### Inhibiting antibodies against TF reverse ROTEM parameters in different fractions

**Figure 5:**
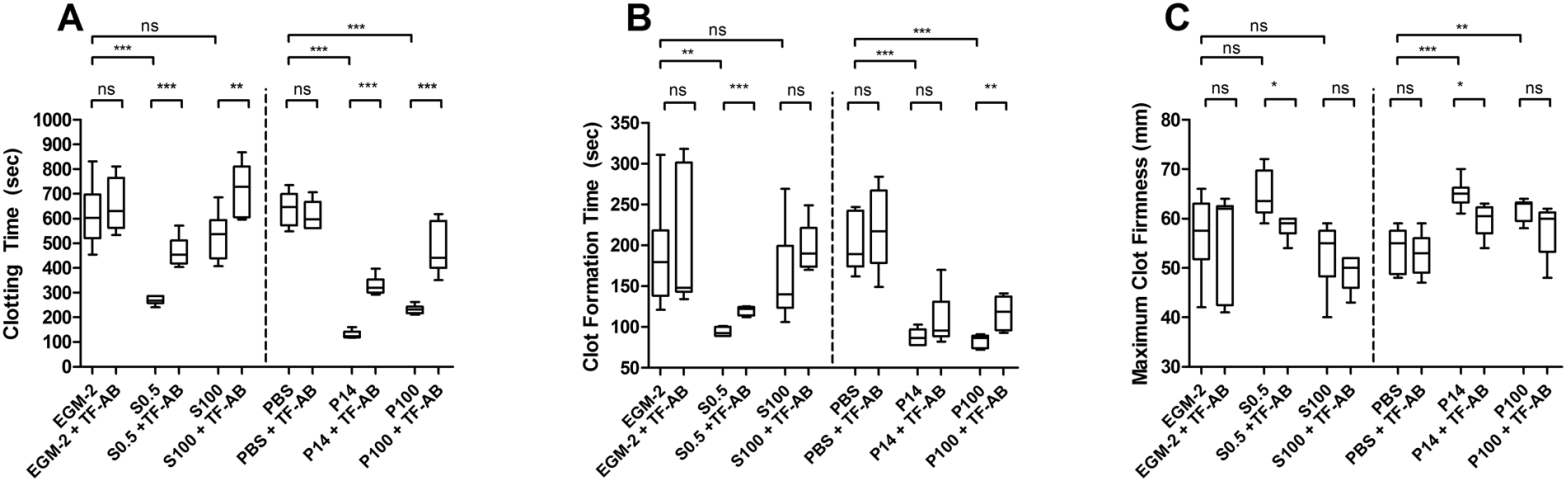
Influence of TNF-α-stimulated EC culture supernatants and concentrated pellets with and without incubation of anti-TF antibody on ROTEM parameters. Different fractions of the supernatants from ECs stimulated with TNF-α for 24 h were collected, additionally exposed to a blocking anti-tissue factor antibody (TF-AB) and 50 µl study samples were added to 300 µl fresh whole blood and subjected to ROTEM analysis. EGM-2 = endothelial growth medium-2; PBS = phosphate buffered saline; S0.5 = cell free culture supernatant; P14 = pellet containing larger vesicles; P100 = pellet containing smaller vesicles; S100 = vesicle free supernatant; ns = not significant; *p < 0.05; **p < 0.01; ***p < 0.001; Box-and-whisker plots (min, max, 25th–75th percentile, median). n = 4–6 measurements.

To investigate the role of membrane-bound TF on CT, CFT and MCF, the samples obtained by differential centrifugation were incubated with inhibitory anti-TF antibody in advance of the ROTEM analysis (Fig. 5). We did not detect any significant influence of this antibody on CT, CFT or MCF in either of the controls (EGM-2 and PBS). Furthermore, we confirmed the significant reduction of CT in the fractions S0.5, P14 and P100 compared to the corresponding controls (Fig. 5A). Exposure of those fractions to anti-TF antibody significantly increased CT again. Although the largely vesicle-free S100 fraction did not significantly reduce CT compared to the corresponding control the anti-TF antibody increased CT in this sample, suggesting that S100 contains non-cell-associated TF, which is not bound on EVs. When analysing the CFT data, we found significant reversions by anti-TF antibody in the S0.5 as well as in the P100 fractions. In contrast, when testing P14 and S100 fractions, the anti-TF-antibody did not exhibit significant differences (Fig. 5B). Finally, the MCF parameter significantly changed in the S0.5 and in the P14 fractions exposed to anti-TF antibody compared to untreated fractions. However, when employing the S100 and P100 samples, no significant differences were measured with anti-TF antibody (Fig. 5C).

### Fractions S0.5, P14 and P100 contain different amounts of active TF

**Figure 6:**
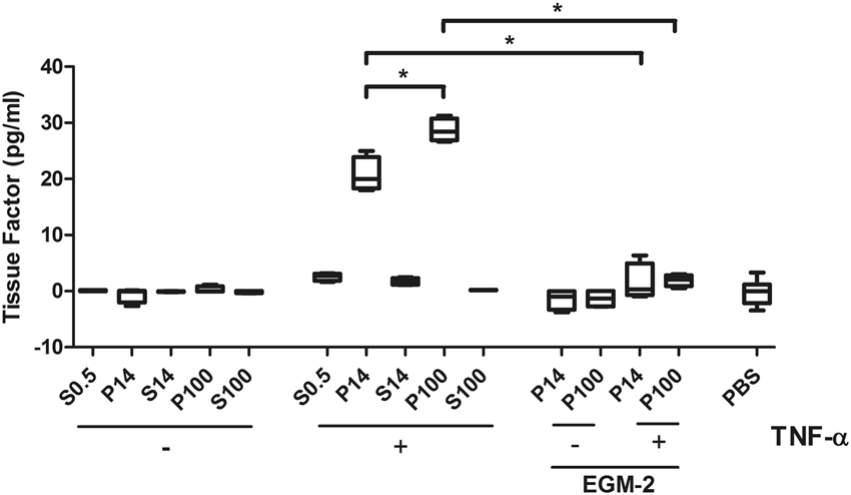
Influence of stimulated endothelial cell culture supernatants and concentrated pellets on a chromogenic assay for the detection of vesicle-bound active tissue factor TF activity was measured in study samples. Supernatants from ECs from at least two different donors that were stimulated with TNF-α for 24 h were collected and subjected to centrifugation steps as illustrated in Fig. 1. PBS = phosphate buffered saline; EGM-2 = endothelial cell growth media-2; S0.5 = cell free culture supernatant; P14 = pellet containing larger vesicles; P100 = pellet containing smaller vesicles; S100 = vesicle free supernatant; Box-and-whisker plots (min, max, 25th–75th percentile, median); *p < 0.05.

Next, we analysed active TF in the fractions obtained by differential centrifugation. Fractions S0.5, P14, S100 and P100 were subjected to TF activity analysis (Fig. 6). We found no TF activity in unstimulated and control samples (PBS and EGM-2) or in the essentially vesicle-free S100 fraction. In contrast, TNF-α-stimulated P14 and P100 samples contained active TF with ~20 pg/ml and ~28 pg/ml, respectively. These results suggest that EVs obtained after the centrifugation steps contain active TF.

## Discussion

In this study we show that EC-derived, TF-bearing EVs can be isolated by differential centrifugation and their biological activity can be measured with thromboelastometry. Pellets obtained from 14,000× g (P14) and 100,000× g (P100) centrifuged supernatant of stimulated endothelial cells exerted a clear procoagulant pattern in fresh whole blood from healthy volunteers. This effect could be partly reversed by an anti-TF antibody, suggestive of a prominent role for TF as a procoagulant stimulus of EVs. The involvement of extracellular vesicle-bound TF could be confirmed by a chromogenic TF activity test. A major aim of the current study was to evaluate which vesicle subtype in TNF-α-stimulated endothelial cell culture supernatants primarily contributed to the observed changes in thromboelastometry. Three elementary subpopulations of EVs have been defined based on their diameter and biogenesis: exosomes (30–150 nm), shedding microvesicles (<1000 nm) and apoptotic bodies (>1000 nm). In contrast to free circulating macromolecules such as hormones, larger EVs can be enriched by centrifugation at 14,000× g (P14) and smaller EVs at 100,000× g (P100). However, several centrifugation protocols exist as particle sedimentation is dependent on the respective particle density^7^. Here, we could show that large particles can be successfully depleted from P100 fractions using our centrifugation protocol. However, the concentration of small particles is equal in both P14 and P100 fractions indicating that small particles already pelleted at 14,000× g with a rotor *k*-factor of 830. Indeed, it has been shown that exosomes can already be pelleted at low g-forces and high *k*-factors and that pelleting efficiency differs greatly among cell types^7^. Nevertheless, we cannot rule out that small particles may have aggregated due to the high centrifugation speed resulting in an increase of size and decrease of concentration^19^. Still, in our functional assays particles obtained from the P14 pellet have been shown to exhibit the strongest biological effect.

Each vesicle-rich pellet was added to fresh whole blood to investigate the isolated influence of EVs on the dynamics of the blood clotting process. In contrast to the control samples using medium only, the supernatant samples of TNF-α-stimulated HUVEC caused a strong CT reduction when added to fresh whole blood. Using differential centrifugation we could provide evidence that for the most part EVs are involved in the CT reduction as the S100 containing soluble factors did not mediate CT reduction. The native, non-activated viscoelastic assay (such as NATEM used in this study) has been proposed to be the most sensitive viscoelastic test for detecting endogenous TF^20, 21^. Although TF influences the speed of clot formation and clot strength (such as CFT and MCF) to some extent, it is the clot initiation (expressed by the CT) that is most markedly altered by the addition of TF in otherwise non-activated viscoelastic assays^22–24^. This is in accordance with the results of the current study, where relatively small amounts of TF significantly affected CT but did not consistently affect CFT and MCF. In whole blood assays, CFT and MCF are primarily dependent on platelet contribution and fibrin polymerisation^25, 26^. However, it has been shown that higher levels of TF activity such as in P14 in our study may also influence clot formation/strength to some extent.

To investigate the role of TF in that context, an anti-TF antibody was successfully used to inhibt its activity.

To confirm that EC-derived and EV-bound TF is most likely the reason for the acceleration of coagulation in thromboelastometry, a chromogenic TF activity assay was performed. This assay is specific to membrane-bound TF, since negatively charged surfaces, such as EVs, are necessary to activate TF. This assay confirmed that EV-bound TF from TNF-α-stimulated endothelial cells could not be detected in the mainly vesicle-free S100 but a high concentration of active TF could be detected in P14. Similar concentrations of active TF could be determined in the P100 fraction as compared to the original supernatant S0.5. The control sample with the medium EGM-2, which is known to contain extracellular vesicles itself, did not show TF activity. These results also confirmed that higher TF activities are in accordance with shorter clotting times in thromboelastometry.

The remaining procoagulant activity of P100 raises the issue that small particles obtained from TNF-α-stimulated HUVEC may contribute to TF-dependent coagulation in our system. However, we and others did not observe a difference in particle count and size of P100 pellets after TNF-α-stimulation of endothelial cells^27^. Furthermore, a study by de Jong OG *et al*. suggests that TNF-α-exosome-driven changes are mainly on the proteome/transcriptome level when vesicles are taken up by host cells^27^. Due to the time frame of our ROTEM experiments (<60 minutes) we excluded any potential influence of exosomes in our setting. Indeed, it has been shown that only the microparticle fraction isolated from the blood of healthy individuals leads to a clotting time reduction while exosomes isolated from the same sample showed no effect on coagulation^28^. Here, we characterised the particle fraction having the strongest biological effect in our functional assay and demonstrate that this effect is at least in part dependent on TF. Furthermore, using flow cytometry we were able to co-detect Annexin V on TF-positive particles, which is both a marker for EVs and an important indicator for procoagulant activity. We additionally provide data on Annexin V-positive but TF-negative particles and *vice versa* indicating that many particles are secreted which are positive for only one of these markers. However, a significant increase upon TNF-α stimulation could be detected in P14 samples regardless of whether particles are single- or double-positive suggesting that particle-bound TF was detected using our flow cytometry protocol.

Nevertheless, we see some limitations in our study. Using anti-TF antibody we did not observe a full but rather a partial reversion of the reduction of CT. This could be due to stochiometrical reasons, in which higher amounts of inhibiting antibody could have led to a complete reversion. On the other hand, other cytokines/mediators than TF or EVs themselves (due to their negatively charged surface) might underlie this effect as well. Nevertheless, CT-reversion in P14 and P100 indicate a potential role of TF-bearing EVs, the existence of which has been published previously^5^. Furthermore, the method of ultracentrifugation (as with other methods also) does not fully separate but rather enriches EVs of different sizes, thus making exact quantifications impossible. We are also aware that protein aggregates are spun down together with vesicular components when exposing supernatants to this magnitude of forces. Additionally, with the use of anti-TF antibody the largely vesicle-free S100 fraction also showed a CT-reversion. This could be attributed to the inhibition of non-cell-associated TF, which is also present in the supernatant of endothelial cells and most likely representing a secreted alternatively spliced form of TF^29^. In addition, besides the use of HUVEC as the “golden standard”, microvascular endothelial cells have been shown to be a physiologically relevant endothelial subtype when it comes to cytokine-triggered TF expression^30^. Moreover, HUVEC are derived from venous origin, and different results might be obtained when using ECs from arterial vascular beds. Finally, although we understand that the transition is fluent, we defined the fractions as either large (P14) or small (P100) and did not further fractionate the initial supernatant samples for the sake of clarity.

In summary, this study provides evidence for the existence and the pro-coagulant activity of TF- bearing EVs, collected from TNF-α-stimulated endothelial cell culture supernatants. EVs of different sizes were separated by ultracentrifugation and their activity was shown by thromboelastometry and a chromogenic TF activity assay. As TF-bearing EVs from endothelial cells may play an important role in cardiovascular disease further research is warranted in order to identify the mechanisms involved and to elucidate the role of procoagulant EVs as potential drug targets in various clinical conditions.

## Methods

### Cell Culture

The experiments were conducted in accordance with the local guidelines and were approved by local ethics committees of the state of Upper Austria and of the AUVA with written informed consent by the donors (ethics committee vote #200). Cells were either isolated according to established protocols^31^ or purchased from Lonza (Walkersville, MD, USA). HUVECs were grown in EGM-2 (Lonza, Walkersville, MD, USA) containing 5% fetal calf serum (FCS) at 37 °C and 5% CO_2_. The cells were tested for their pro-inflammatory responsiveness by stimulating them with 10 ng/ml TNF-α (Sigma Aldrich, St. Louis, MO, USA) for 4 h. Subsequently, cells were tested for their ELAM-1 expression by flow cytometry (data not shown). All cell types used in this study showed strong inducibility of this surface marker. For the isolation of EVs, conditioned medium was collected from confluent HUVEC after stimulation with 10 ng/ml TNF-α for 24 h.

### Isolation of different EVs from conditioned medium

For EV collection from the cell culture supernatants, serial centrifugation steps were conducted (Fig. 1). EVs were isolated by differential centrifugation to obtain vesicles of various size-range. 1.5 ml of the supernatant from approximately 5 × 10^5^ cells were centrifuged at 500× g and 15 minutes (Eppendorf F-45-24-11 rotor, rotor *k*-factor 830, 4 °C) for cell removal resulting in the so called supernatant S0.5. The fraction S0.5 contained both larger and smaller EVs. This S0.5 was then centrifuged at 14,000× g for 15 minutes (Eppendorf F-45-24-11 rotor, rotor *k*-factor 830, 4 °C) to collect the pellet P14, which was assumed to contain mainly larger EVs, enriched in shedding microvesicles but also apoptotic bodies and cell debris. The remaining supernatant was subjected to an additional centrifugation step of 100,000× g for 60 minutes (Sorvall S45A Angle rotor, rotor *k*-factor 67, 4 °C) to obtain the so called pellet P100 enriched in exosomes. The residual supernatant S100 was intended to be mostly vesicle-free. Both pellets P14 and P100 were gently resuspended in 50 µl phosphate buffered saline (PBS). Unconditioned medium alone (EGM-2 containing 5% non-EV-depleted FCS, incubated at 37 °C and 5% CO_2_) was used as a negative control in all experiments and equally processed for EV isolation as described above.

### Nanoparticle tracking analysis (NTA)

The size and concentration of isolated particles was determined with the Zetaview PMX 110 V3.0 particle analyser (Particle Metrix GmbH, Meerbusch, Germany) as described previously^32^. Briefly, samples were diluted 1:250 in sterile-filtered 1x PBS prior to staining for flow cytometry, injected into the device and videos were recorded for 60 seconds at two positions of the measurement cell. Camera sensitivity was determined automatically for each sample. Videos were analysed using ZetaView 8.02.31 software.

### Flow cytometry

After stimulation of HUVEC with 10 ng/ml TNF-α for 24 hours, supernatants were removed, processed and cells were detached using Accutase solution (Sigma-Aldrich, St. Louis, MO, USA). Cells were kept in suspension on ice during staining with 5 µg/ml anti-TF antibody (American Diagnostica, Lexington, USA). After 45 minutes of incubation, bound antibody was detected by incubation with 10 µg/ml anti-mouse Alexa Fluor® 488 (Thermo Fisher, Eugene, Oregon, USA) for 45 minutes on ice. To stain isolated EVs, the particle pellets P14 and P100 were incubated with 10 µg/ml anti-TF antibody for 30 minutes on ice, washed with 1 ml PBS/BSA (1%) and centrifuged at 16,100× g for 20 minutes (Eppendorf F-45-24-11 rotor, rotor *k*-factor 830, 4 °C). 1 ml supernatant was discarded and the bound antibody detected by incubation with 20 µg/ml anti-mouse Alexa Fluor® 488 for 30 minutes on ice. The particle suspension was again washed and centrifuged before 1 ml supernatant was discarded and 2 µl Annexin V (eBioscience Inc, San Diego, USA) in a total of 300 µl 1x Annexin V Binding Buffer were added to the remaining 100 µl. This staining protocol does not result in loss of Annexin V-positive particles in P14 and P100 fractions (Supp. Fig. 2). After 15 min of incubation on ice, all samples were analysed on a Beckman Coulter CytoFlex flow cytometer (Beckman Coulter Inc., Brea, USA) and particle counts present in the total sample volume (400 µl) were calculated. No swarm signals were detected using this sample preparation protocol (Supp. Fig. 3A). For EV analysis, a slow flow rate (10 µl/min) and 60-second stop time were chosen. We demonstrated the flow rate stability with 1000 nm silica calibration beads (Suppl. Fig. 3B). Furthermore, using 100, 500 and 1000 nm silica beads as gating parameters, microparticles (MP) were defined as particles smaller than 1000 nm that can still be distinguished from noise (Supp. Fig. 1). Additionally, in order to get rid of antibody aggregates all antibodies were centrifuged at 16,100× g for 5 minutes prior to use, and the sample line of the flow cytometer was cleaned with distilled water at a fast flow rate (60 µl/min) for 2 minutes between each sample measurement. Flow cytometry was repeated two times using different endothelial cell donors.

### Inhibition with anti-TF-antibody

To prove the influence of TF on potential pro-coagulant effects, all samples (S0.5, P14, S100, P100) were incubated with 1 µg anti-TF (0.5 mg/ml, American Diagnostica, Lexington, USA) for 30 min at 37 °C.

### Thromboelastometry (ROTEM)

ROTEM (TEM Innovation, Munich, Germany) is a viscoelastic method for the analysis of the clotting dynamics during whole blood coagulation^33^. ROTEM uses a rotating pin that is vertically immersed into a prewarmed cup containing the blood sample. Coagulation of the citrated blood sample is initiated by recalcification and is detectable by the reduced rotation range of the pin due to the formation of a fibrin clot between the pin and the cup wall. The generated signal is converted into a curve that indicates the time of clotting initiation, the speed of clot formation, the clot strength as well as the clot quality/lysis^33, 34^. In clinical diagnostic situations, ROTEM assays are usually activated via contact activation (intrinsic pathway, INTEM) or with tissue factor (extrinsic pathway, EXTEM). However, to be sensitive to minimal amounts of TF, the initially nonactivated assay NATEM was used in the current study^22^. Briefly, 20 µl of CaCl_2_ (200 mmol/l) were added to 300 µl of citrated blood (freshly taken blood from healthy human volunteers anticoagulated with 3.2% trisodium citrate in a 9:1 ratio, respectively) followed by addition of 50 µl of the study sample (either S0.5, P14, S100 or P100) before the rotating pin was immersed into the mixture of blood and study sample. On average, 61906 and 1246 Annexin V- and TF-double positive particles obtained from P14 and P100 fractions of TNF-α-stimulated HUVEC, respectively, were added to the assay matrix in ROTEM. Particles were always isolated from a confluent well of a 6-well plate containing 2 ml of growth medium. The parameters clotting time (CT), clot formation time (CFT) and maximum clot firmness (MCF) were evaluated. ROTEM experiments were repeated between four and six times with at least two different HUVEC donors.

### TF activity test

TF activity of EVs dervied from HUVEC stimulated with and without TNF-α was assessed as described elsewhere^35^. Briefly, 50 µl of all centrifugation samples (S0.5, P14, S14, P100, S100) were incubated with either mouse anti-human TF antibody (American Diagnostica, Lexington, USA) or mouse IgG-control (Sigma Aldrich, St. Louis, MO, USA) for 15 min at room temperature. Subsequently, samples were incubated with 50 µl of HBSA containing 10 mM CaCl_2_, 10 nM Factor VIIa (CoaChrom Diagnostica GmbH, Austria) and 150 nM Factor X (CoaChrom Diagnostica GmbH, Austria) for 2 hours at room temperature. The reaction was stopped with 25 µl HBSA containing 25 mM EDTA and samples were incubated with 25 µl chromogenic substrate (Pefachrome® FXa-8595, Pentapharm, Basel, Switzerland) for 15 min at 37 °C, and absorbance was measured at a wavelength of 405 nm. Samples were measured in duplicates and EGM-2, which was not incubated with cells, as well as PBS served as negative controls. TF activity was calculated by reference to a standard curve generated using relipidated rh TF (Innovin®, Siemens Healthcare Diagnostics, Austria). The TF-dependent FXa generation was determined by subtracting the amount of FXa generated in the presence of anti-TF antibody from the amount of FXa generated in the presence of the isotype control antibody.

### Statistical Analysis

Data were analysed with GraphPad Prism 5 (GraphPad Softward Inc., La Jolla, CA, USA). Based on distribution of data analysed with a Kolmogorov-Smirnov-test a Student t-test or a Mann-Whitney test were used to compare mean differences between corresponding groups. Fold changes were analysed by a Wilcoxon matched-pair signed rank test. P-values < 0.05 were considered to be significant.

## Electronic supplementary material

Supp Info
