# Supplementary material for: Endothelial Cell-derived Extracellular Vesicles Size-dependently Exert Procoagulant Activity Detected by Thromboelastometry
Source: Sci Rep. 2017 Jun 16;7:3707. doi: 10.1038/s41598-017-03159-0 (PMC5473891; doi:10.1038/s41598-017-03159-0)
Supplement: Supplementary file 1 — Supp Info [file 41598_2017_3159_MOESM1_ESM.doc]

**Supplementary Information**

**Endothelial Cell-derived Extracellular Vesicles Size-dependently Exert Procoagulatory Activity Detected by Thromboelastometry**

Wolfgang Holnthoner, Cornelia Bonstingl, Carina Hromada, Severin Muehleder, Johannes Zipperle, Stefan Stojkovic Heinz Redl, Johann Wojta, Herbert Schöchl, Johannes Grillari, Sylvia Weilner, Christoph J. Schlimp

**Supplementary Figure 1:** **Use of calibration beads to adjust flow cytometry parameters.**

Silica beads of defined size (100, 500 and 1000 nm) were used to define the microparticle gate that includes particles smaller than 1000 nm that can still be distinguished from the background signal.
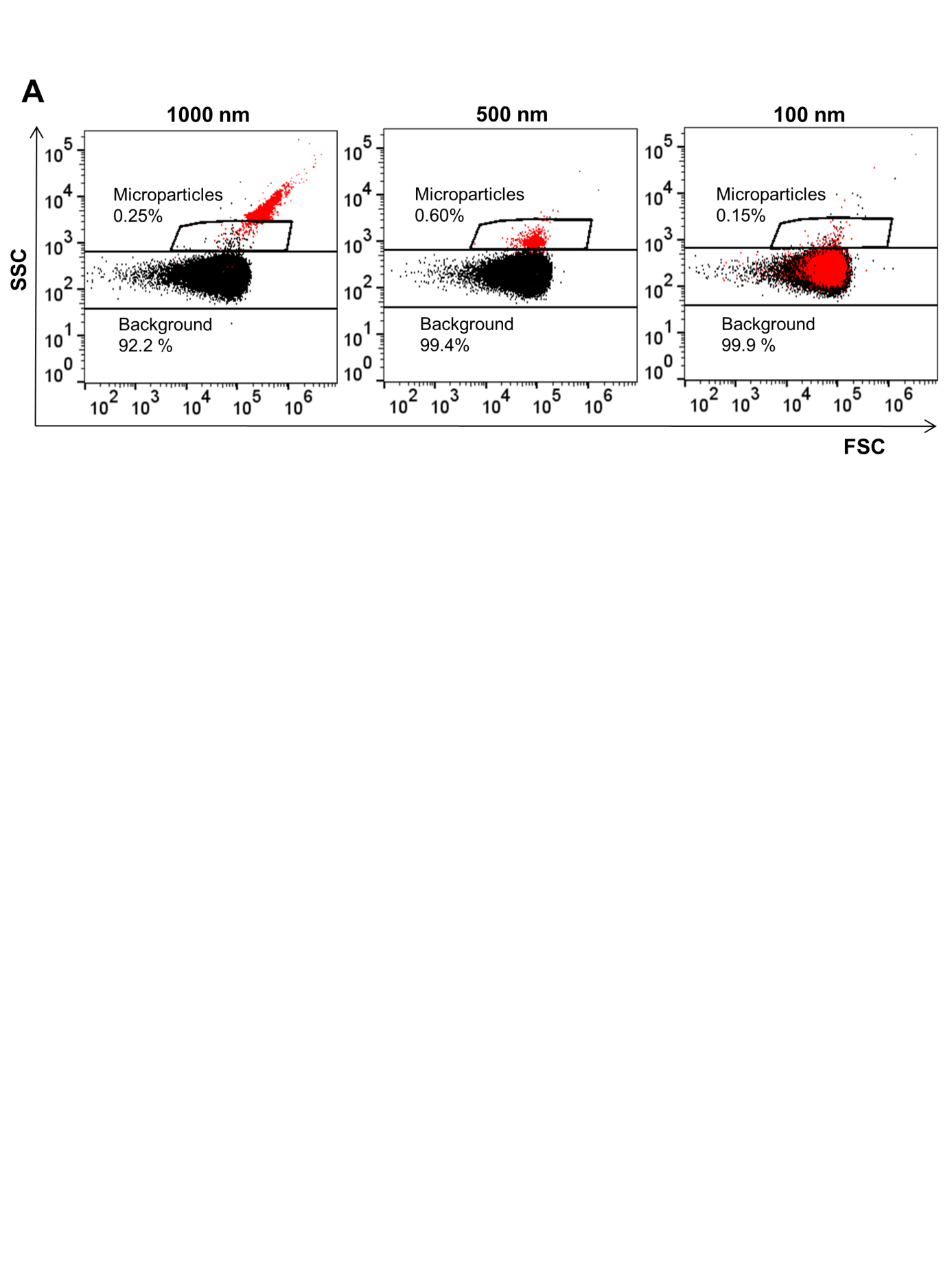


**Supplementary Figure 2: Influence of 16,100 x g centrifugation on detection of Annexin V^+^ particles by flow cytometry.**

Particles in P14 fractions were isolated from TNF-α-stimulated cells and either subjected to the centrifugation steps and stained according to the protocol described in the methods section or stained directly without centrifugation. Analysis of fold changes of Annexin V^+^ particles indicates that the additional centrifugation steps do not influence the particle yield in flow cytometry. Values are from two separate experiments using two HUVEC donors.


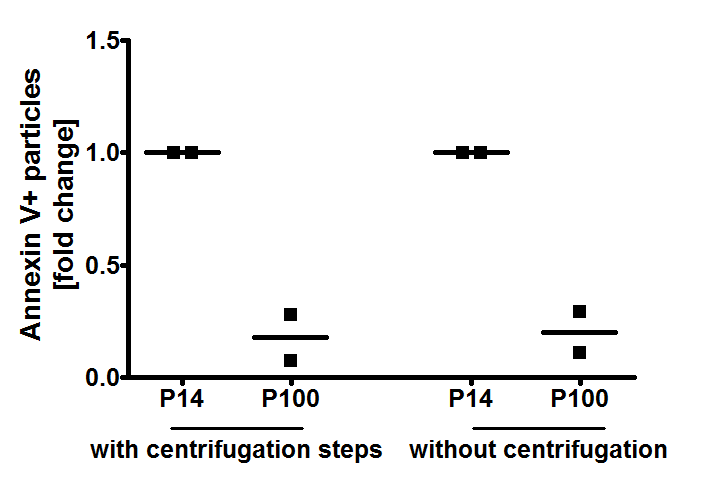


**Supplementary figure 3: Detection of swarm signals and flow rate calibration.**

Serial dilution of stained P14 isolates from TNF-α
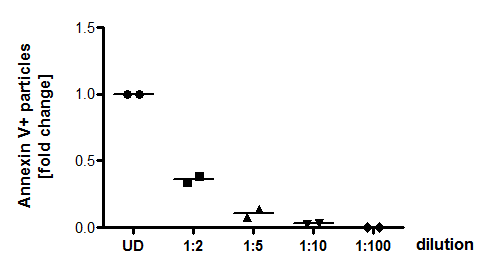
-stimulated cells was performed in PBS to determine if swarm signals are detected. No swarm effect could be observed indicating that single particles were detected using the established flow cytometry protocol. Values are from one experiment and donor (A). Silica calibration beads were used to count events/µl in a fixed gate for a 60 second measurement period to demonstrate the stability of the flow rate (B).

**A**

**B**


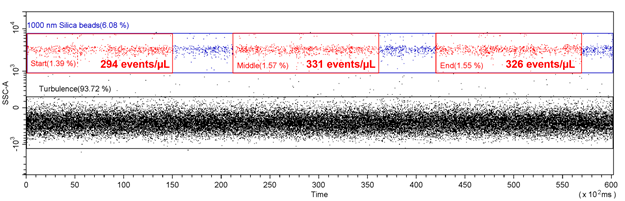


**Supplementary figure 4: Concentration and size measurement using NTA.** Total particle count (A) and size (B) of particles detected in all fractions described. Values were obtained from separate experiments using at least two different donors. n ≤ 5. **p<0.01.

**A**


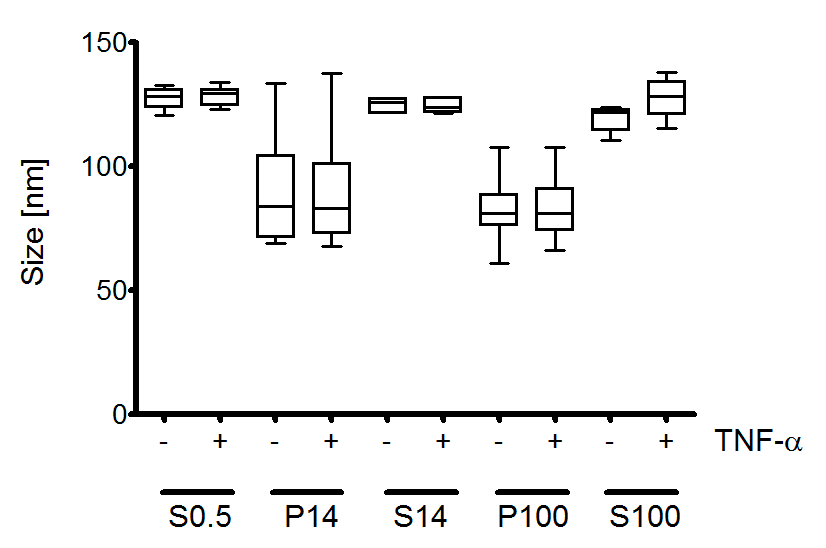

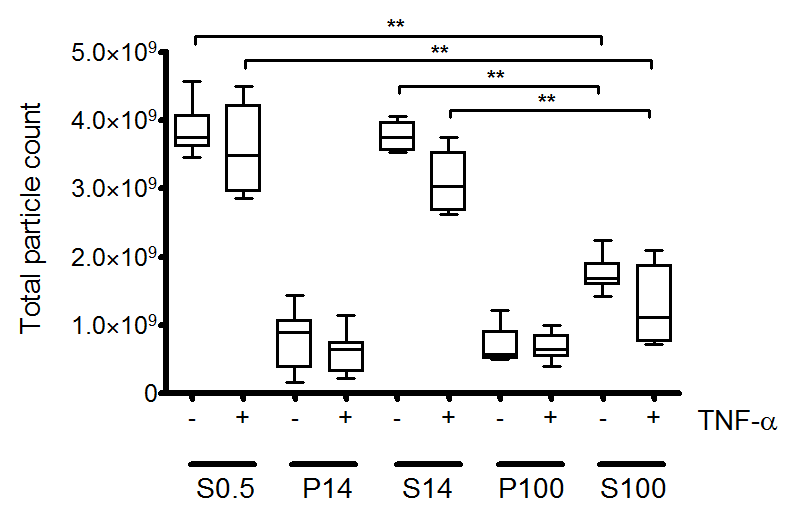


**B**
